# Contemporary challenges for a curriculum to foster interest in surgical careers: a multicentric study on the evolving needs of female medical students to consider a career as a surgeon in Germany

**DOI:** 10.1186/s12909-026-09247-y

**Published:** 2026-07-03

**Authors:** Riko Kelter, Sebastian Dango

**Affiliations:** 1 Institute of Medical Statistics and Computational Biology (IMSB), Faculty of Medicine, University of Cologne, Robert-Koch-Str. 10, Cologne, Northrhine-Westphalia 50931 Germany; 2 Department of Visceral, Bariatric, and Metabolic Surgery, Klinikum Siegen, Weidenauer Str. 76, Siegen, Northrhine-Westphalia 57076 Germany; 3 Department of Visceral, Thoracic and Vascular Surgery, Philipps-University Marburg, Baldingerstrasse 1, Marburg, Hesse 35043 Germany

**Keywords:** Women in surgery, Surgical curriculum, Surgical careers, Gender discrimination in surgery

## Abstract

Early obstacles to career progression of women in surgery present a major challenge in a variety of surgical subspecialties. Although there is a variety of literature on the attitudes and needs of female residents and trainees to foster interest in a career as a surgeon, there is a lack of research about the perspectives of female medical students about a possible career as a surgeon. In this paper, we report the results of a multicentric study on the evolving needs of female medical students to consider a career as a surgeon in Germany. We confirm several results mentioned in the available literature on the topic, but also add novel insights, in particular, which attitudes and obstacles already shape earlier than during trainee programmes or residency, namely, during medical school. Our results indicate that a curriculum to foster interest in surgical careers has to incorporate three core ingredients: (1) Effective concepts against gender discrimination, (2) measures to work towards more flexible work times and better work-life-balance of female surgeons and (3) an increase in female role models and mentoring programmes, tailored specifically for female medical students.

**Supplementary Information:**

The online version contains supplementary material available at 10.1186/s12909-026-09247-y.

## Background

Early obstacles to career progression of women in surgery present a major challenge in a variety of countries [1]. According to Ferrari et al. [1], different impediments hinder surgical career progression for women, with notable consequences on burnout and attrition. Stephens et al. [2] stress that despite women often composing half of current medical school classes, surgical specialties still struggle to attract and retain women. Even once successful recruited into training, female surgeons go on to face gender bias and multiple obstacles to career advancement. Such obstacles include lower rates of surgical residency completion, board certification, and professional advancement [2]. As a consequence, Gender inequality in medicine has drawn attention in the literature, in particular, the status of women in surgery [1–3].

According to Stephens et al. [2], obstacles to career development for female surgeons include residency and fellowship support, a lack of mentorship and sponsorship, a lack of work-life balance, and no pay equity. Ferrari et al. [1] in a scoping review analyzed more than 120 studies investigating barriers to career progression of women in surgery and solutions to improve them. Of the articles included, 22 (18%) focused on factors that affect the pursuit of a surgical career, such as surgical work hours and limited time for outside interests. Roughly half of the studies, 55 (46%) analyzed the main barriers that exist during surgical residency and fellowship training, such as discrimination and sexual harassment. About a fourth of the studies, 27 (23%) investigated barriers to career advancement. These included – similar to the results reported by Stephens et al. [2] – heavy workloads, ineffective mentorship, unclear expectations for advancement of a career, inequality in pay or, again, work-home conflicts. The number of studies reporting on possible solutions was quite small: Only 8 (6.5%) articles reported on the role of effective mentorship to support career advancement and to provide moral support and 8 (6.5%) on the emerging role of social media for networking [1].

The role of mentorship in fostering interest in a career as a surgeon is also stressed by Case et al. [4]: Various studies agree that although female surgeons are likely to express a desire for a same-sex mentor, there is a notable lack of female mentors available to them. Thus, Case et al. [4] came to the conclusion that effective mentoring from multiple mentors, preferably some of which identify as female, may improve career advancement in female surgical subspecialists and promote gender equality in these fields.

When it comes to regional or subtype differences between effective curricula to foster interest in surgical careers, the situation becomes even more complicated: Ruparell et al. [5] studied motivators and deterrents for early career female doctors applying to surgical training programmes in the UK National Health Services. A total of 44 out of 100 questionnaire respondents in the study ranked early exposure to surgery as the most influential motivator, while 43% chose work-life balance as the greatest deterrent and 33% suggested that mentoring schemes to encourage women to apply to core surgical training programmes would be helpful. This is in line with the findings of Stephens et al. [2] and Ferrari et al. [1].

Heisler et al. [3] focussed on obstetrician-gynecologists, and unlike surgical specialties that remain predominantly male, the majority of obstetrician-gynecologists have been women for nearly a decade. Women have composed the majority of trainees since the 1990 s, but still, despite a critical mass of women, biases related to gender persist in the field.

Barnes et al. [6] conducted a mixed-methods study, where the aim was to describe female surgeons experiences with gender bias and microaggressions in the workplace during residency and fellowship training. The goal of the study was to understand if differences exist in the experiences of trainees in male-dominant vs female-dominant surgical specialties. The conclusion of Barnes et al. [6] is that female surgical trainees continue to experience gender bias. According to Barnes et al. [6], a culture of sexism leads to physical and social adaptations to fit into the role of the surgeon. Participants of the study expressed significant effort to sustain this level of adaptation, which ultimately led to fatigue and creation of resilience mechanisms. The study demonstrated that the environment in which a trainee operates (male-dominant vs female-dominant) significantly impacts their experience. Those experiencing more bias were less likely to recommend their specialty and reported plans to leave medicine earlier. Barnes et al. [6] recommend that a culture change across institutions and system-level interventions is necessary to create meaningful and sustainable change that improves the experience of female surgical trainees.

Bellini et al. [7] reached similar findings in the UK and Ireland, and Benzil et al. [8] studied the situation in neurosurgery and came to the conclusion that *“the consistently low numbers of women in neurosurgery training programs and in the workplace results in a dearth of female role models for the mentoring of residents and junior faculty/practitioners. This lack of guidance contributes to perpetuation of barriers to women considering careers in neurosurgery, and to the lack of professional advancement experienced by women already in the field.”* ([8], p. 1)

Taking stock, there are several well-established factors which can be regarded as obstacles for female trainees and residents to consider or pursue a career as a surgeon. Prime examples of these obstacles are heavy work-load, aspects such as family planning, discrimination, the lack of role models or mentors and a male-dominant culture. While there might even be additional factors not reported of studied in detail in the literature, all of the studies we found focus on trainees or residents instead of medical students. Also, none of the studies dealt with female medical students in Germany. Regarding the aspects which constitute obstacles for considering a career as a surgeon, other factors such as exposure to specialties during medical clerkships and sponsorships might be relevant for medical students, in contrast to trainees of residents who already graduated and are more experienced. It can therefore be expected that while some perceptions will be shared by medical students and trainees or residents, others could differ substantially.

### Outline

The current state of research reported in the literature therefore unfolds as follows: First, although there is plenty of research regarding the challenges for female medical students and trainees when considering a career as a surgeon, the current literature focusses mostly on specific subdomains of surgery or on trainees and residents. Multiple studies exclude the perspective of female medical students, although it can be expected that attitudes towards a career in surgery form *early* during medical school, and not only during trainee programs or residency. This is undermined, in particular, by the results of Ruparell et al. [5], according to which about a half of the study participants rated early exposure to surgery as the most influential motivator to consider a later career as a female surgeon. We stress that the study only considered the perspective of trainees and not of medical students. In particular, the majority of german medical students are female with increasing trend, stressing the importance of the topic.

Second, the literature shows a lack of studies which investigate this phenomenon in Germany. Such a study is the first step for the creation and implementation of curricula that meet the evolving needs of surgical trainees and addresses contemporary healthcare challenges which are due to the demographic situation in Germany, mainly an aging society and problems with medical care in rural areas.

As a consequence, in this paper we focus on contemporary challenges for a curriculum to foster interest of female medical students in surgical careers. Therefore, we report the results of a multicentric study on the evolving needs of female medical students to consider a career as a surgeon in Germany.

Our approach has two specifics: First, we focus solely on female medical students and exclude trainees and residents, because attitudes towards surgery supposedly form early during medical school, compare Ruparell et al. [5]. Second, we conduct a multicentric study which investigates female medical students’ attitudes towards motivators and deterrents which could possibly foster a career as a surgeon. The literature reports an abundance of these deterrents and motivators, compare the introduction section above. Here, we concentrate on aspects which have been isolated multiple times in the available literature such as working load and times, the availability of mentors and female rolemodels, and some others. We describe details of our questionnaire below. Also, we provide room for additional input from the participants. This additional input allows to get insights into what could improve interest in a career as a surgeon and remove currently established barriers, in particular, for female medical students in Germany.

## Methods

### Study design

The study was carried out as an online questionnaire at the university of Bonn and Marburg in western Germany. Inclusion criteria for students were a semester larger than 2, so students had to be in their third semester of medical school as a minimal requirement, since in third semester students had already contact with clinical subjects. Also, only female students and no residents or trainees were included in the study, compare Background section. These students had no mentor and at least one surgical rotation of four weeks in the surgical clinic.

The full questionnaire in original German wording is available as a Supplemental file and was translated into english for presentation of the results in the following section.

Questionnaire items were constructed by the human resources department at Klinikum Siegen with the aim to gain insights into contemporary challenges for a curriculum to foster interest in surgical careers in female medical students in Germany. Therefore, several dimensions based on the available literature were considered and the original German questionnaire as well as an english translation are provided in the Supplemental file. The survey itself was developed in an interdisciplinary cooperation between the human resources team and the women’s representative of the clinic, taking into account the available literature. The items of the questionnaire were created based on the primary research question, that is, which aspects could be a contemporary challenge for female medical students to consider a career as a surgeon. The majority of these items overlapped with the ones reported in the literature on trainees or residents, compare the aspects mentioned in the background section (e.g. the role of mentorship, gender discrimination, heavy workloads, work-life balance). Multiple other items were added to gain further information, such as detailed questions about which actions could be taken to improve interest in a career as a female surgeon, the experiences of female medical students during internships in surgery, questions about further resources which would help in considering a career as a surgeon and questions about the aspects of surgery which are highly appealing to female medical students. These further items were not isolated based on the literature but added with the goal to provide insights in addition to the already studied perceptions of female trainees and medical residents, where for the latter we expect that female medical students will share some of these perceptions.

For presentation of the results, the original German wording was translated to english.

The study was not reviewed by an institutional review board (it was submitted to the ethics committee of the University of Marburg, but judged as not necessary to be reviewed formally).

### Statistical analysis

Statistical analysis was carried out using R [9]. For most questionnaire items, simple bar graphs suffice for an exploratory data analysis, which was the primary goal of the study. The means and standard deviations are given numerically and as error bars in these plots. Exceptions are the age and semester of the participants where only means are given. For clarity reasons, we provide counts and percentages for the answers to most questions to make clear how many participants responded to a question. As noted above, most questionnaire items were answered by all participants with very few exceptions, so missing data was no problem in this study.

## Results

In this section, the results of the study are presented and discussed.

In sum, a total of $n=182$ students answered the questionnaire. The questionnaire itself was distributed via the common communication channels of the universities organized by the medical denary. This was the most effective way of communication since all students are included in the communication. Still, as it remains unknown which students read this communication it remains unknown to us how many students were solicited to participate, so we can not provide the percentage of students who participated. Still, the results presented in the next section also demonstrate that the vast majority of students answered all questions, so only very few items have missing data, that is, are not answered by all participants. This shows that students’ interest in the topic is, in general, substantial.

**Fig. 1:**
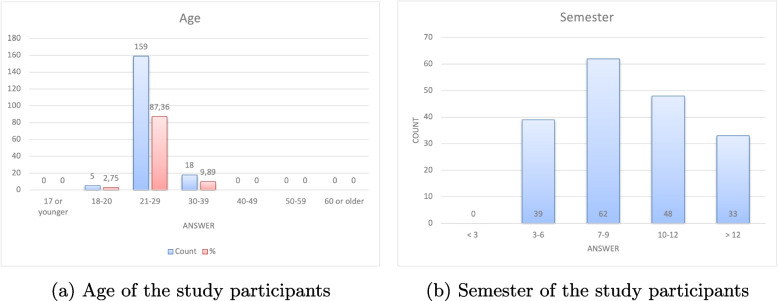
Distribution of age and semester of study participants. Panel (**a**) shows the age, panel (**b**) the semester of study participants

**Fig. 2:**
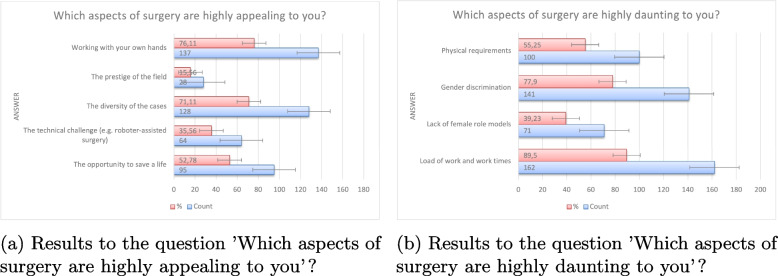
Highly appealing and highly dauunting aspects of surgery for female medical students in Germany. Panel (**a**) shows highly appealing, panel (**b**) highly daunting aspects

**Fig. 3:**
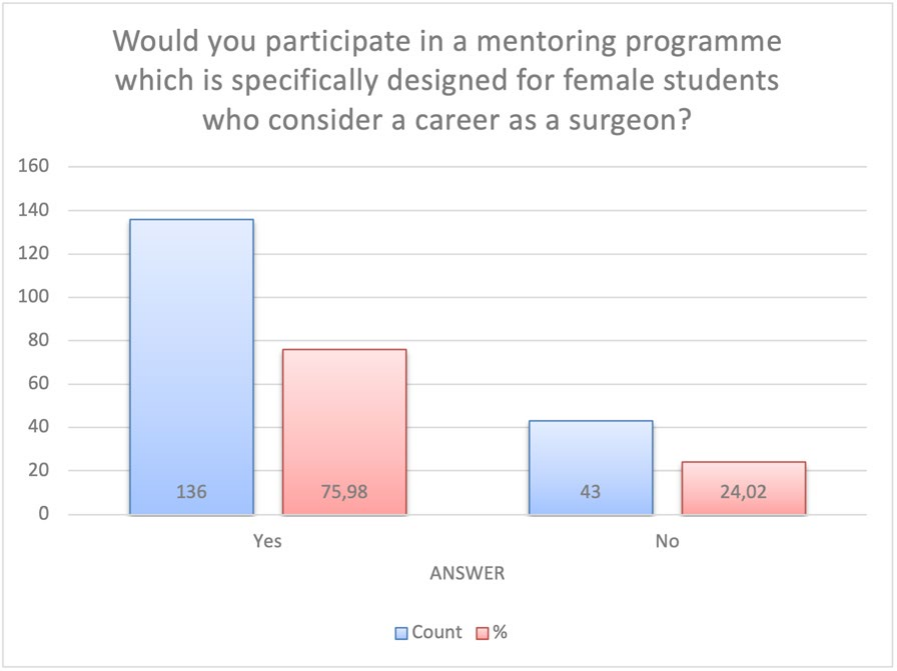
Results to the question ‘Would you participate in a mentoring programme which is specifically designed for female students who consider a career as a surgeon?’

**Fig. 4:**
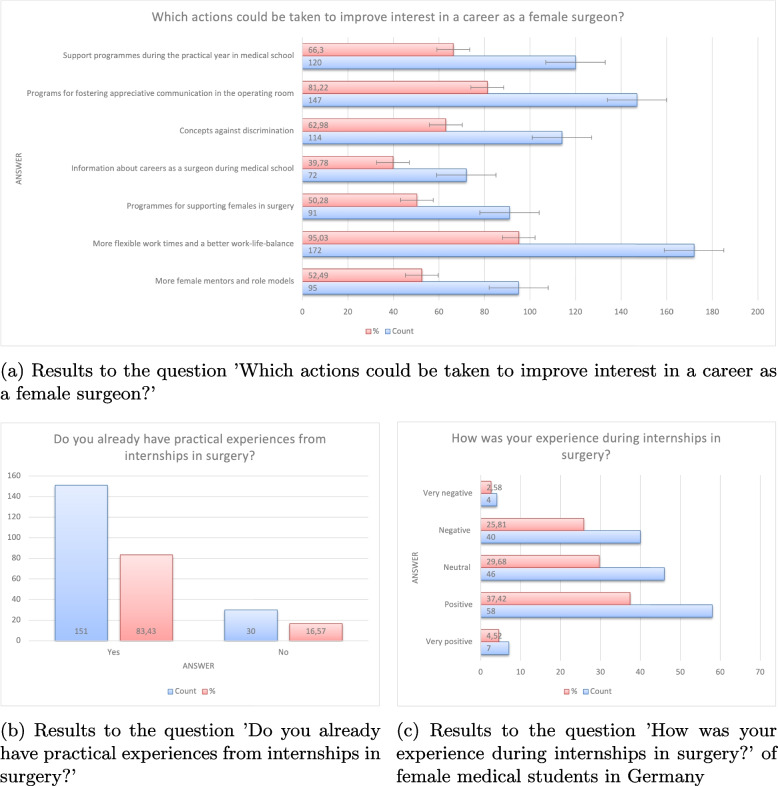
Actions which could improve interest in a career as a female surgeon, percentage of participants who have already practical experiences from internships and experiences during these internships. Panel (**a**) shows which actions could be taken to improve interest in a career as a surgeon, panel (**b**) the distribution of practical experiences of participants in surgery, and panel (**c**) how participants judged these experiences in surgery

**Fig. 5:**
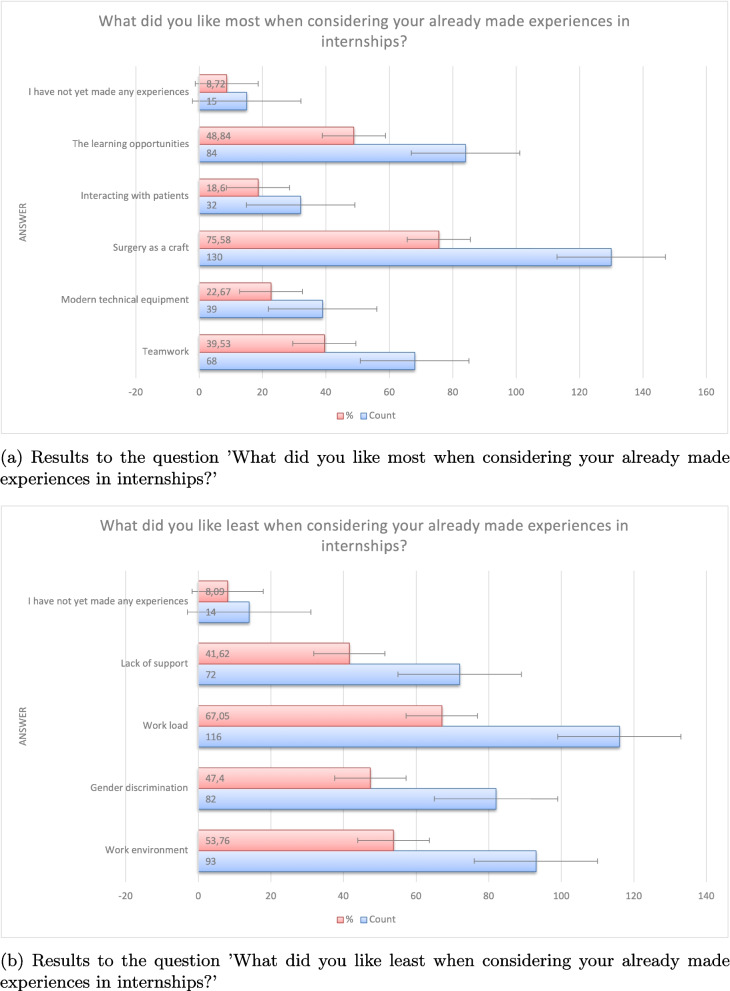
Experiences during internships of female medical students in Germany. Panel (**a**) shows what participants liked most during internships in surgery, panel (**b**) what participants liked least.

Figures 1a, 2, 3, 4 and 5b visualize the answers of female medical students. As shown in Fig. 1a, most participants were between 21 and 29 years old, with 87.36% respectively 159 answers. Participants age ranged from possibly 18 to 39 years, and most participants were in their seventh to ninth semester, compare Fig. 1b. The data show that the vast majority of participants was already in their clinical studies during medical school, which starts at the fifth semester in Germany. Thus, 143 out of 182 participants were in semester seven or higher. Figure 2a and b paint an interesting image about the appealing and daunting aspects female medical students perceive about surgery. As demonstrated in Fig. 2a, to 76.11% it is appealing to work with one’s own hands. While the prestige of the field does not raise much interest, the diversity of the cases (71.11%) and the opportunity to save a life (52.78%) appeal to female medical students. Technical challenges only appeal to about a third of the cohort. Figure 2b demonstrated clearly that gender discrimination is a daunting aspect of surgery for female medical students in Germany: About three out of four students, that is, 77.9% answered positively here. This is even more than the lack of role models (39.23%) or physical requirements (55.25%) and is only surpassed by the load of work and working times (89.5%).

Figure 4a shows the answers of students to the question which actions could be taken to improve interest in a career as a female surgeon. Confirming the impressions from the results shown in Fig. 2b, 95.02% answered that more flexible work times and a better work-life-balance are one action which could improve interest. Programs for fostering appreciative communication in the operating room follows with 81.22%, and support programmes during the practical year in medical school (66.3%). While more female mentors and role models as well as programmes for supporting females in surgery are seen as reasonable actions by about one out of two female medical students, concepts against discrimination are seen as more important (62.98%). This raises the question how to improve communication and anti-discrimination concepts, in particular, in the operating room.

In our study, 151 out of 182 participants had already made practical experiences from internships in surgery, compare Fig. 4b. About three out of four students, that is, 75.98% would participate in a mentoring programme which is specifically designed for female students who consider a career as a surgeon, see Fig. 3, being congruent with the earlier results of Ferrari et al. [1], Stephens et al. [2] and Heisler et al. [3].

Figure 4c shows that most students rate their experience during internships in surgery as positive or at least neutral (37.42% and 29.68%). Only 25.81% rate it as negative, while 2.58% rate is as very negative. Although these latter numbers are still too high, the majority of students seems to make neutral to positive experiences in surgery internships. This is in contrast to the apparent problems with sparking interest in female medical students to consider a career as a surgeon.

Figure 5a and b delve deeper into the issue and show the answers to the questions what students liked most or least when considering their already made experiences in internships. Figure 5a shows that surgery as a craft has a strong appeal to 75.58% of the students. The other aspects are much less positively seen by students, e.g. the learning opportunities (48.84%) or the teamwork (39.53%). Neither modern technical equipment (22.67%) nor the interaction with patients (18.63%) are remembered as positive by the majority of students when faced with the options given in Fig. 5a.

Figure 5b adds to the picture that the work load (67.05%) and work environment (53.76%) are seen as the biggest obstacles for most students. Again, gender discrimination (47.4%) and a lack of support (41.62%) follow with some distance.

We close this section by noting that there are some inconsistencies between the number of students who marked that they have no experiences made yet: In Fig. 5a and b they differ by one person, and in Fig. 4b they differ even further. This makes it hard to clearly tell how many students have made practical experiences precisely, but from the data at least $n=151$ seems to be the minimum, which is still at least 83.43% of students participating in the study.

We now turn to the issue of flexible work times. From the results it is already apparent that a clear issue in Germany seems to be the need for a better work-life-balance and more flexible work times. The work load is perceived as highly negative by most students, presenting a strong deterrent to consider a career as a surgeon.

**Fig. 6:**
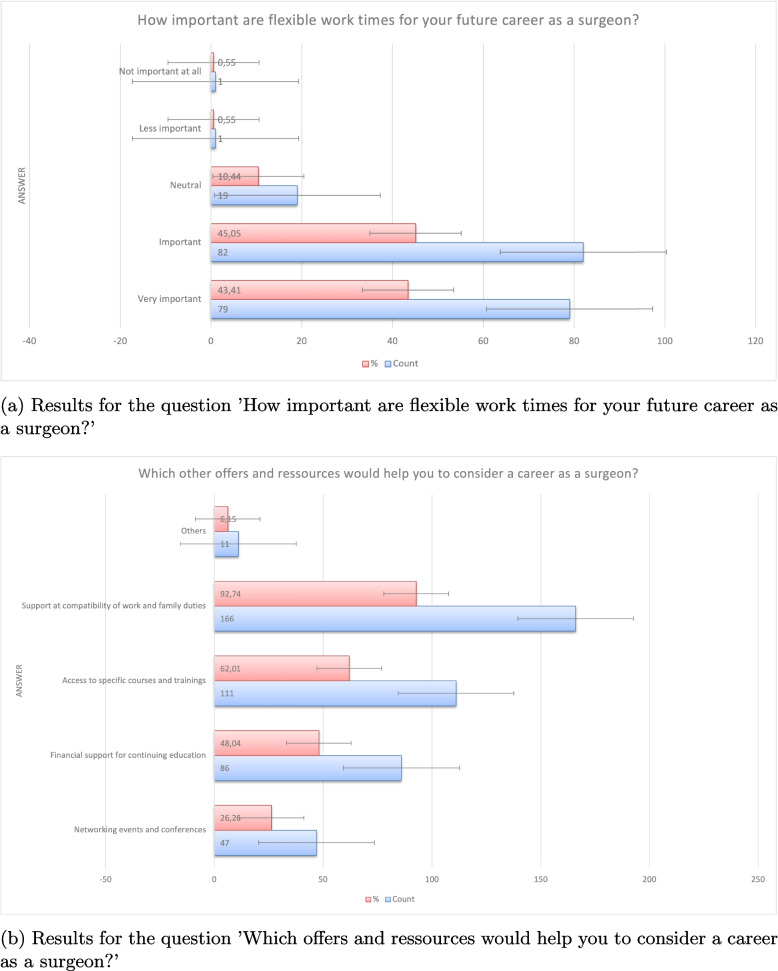
Perspectives on the importance of flexible work times and other ressources to help to consider a career as a surgeon. Panel (**a**) shows how important flexible work times are considered by participants for a possible career as a surgeon, panel (**b**) what other ressources and offers might help in considering a career as a surgeon.

Figure 6a shows that 45.05% rate flexible work times for their future career as a surgeon as important, and 43.41% as very important. Less than one percent rate this factor as less or not important at all. In sum, nearly 9 out of 10 students see this factor as important or very important.

Figure 6b completes this picture by demonstrating that 92.74% of the participants think that support at compatibility of work and family duties would help them to consider a career as a surgeon. This aspect clearly dominates the scene: Access to specific courses and trainings is thought of as helpful by 62.01%, and financial support for continuing education (48.03%), or networking events and conferences (26.26%) resonate much less with the female medical students who participated in the study.

## Discussion

In this section, we discuss the results of the study. In our study, we could confirm earlier results – compare Background section – which postulated that work load and environment contribute to the dearth of females who consider a career as a surgeon. Our results indicate that this phenomenon holds also in Germany. More importantly, our results show for the first time that the impressions and attitudes towards a possible career in surgery form early, as early as from the third semester on. As stressed in Background, most of the literature focusses on residents or trainees. Our results clarify, however, that interventions to raise interest in females to consider a career as a surgeon must start much earlier, ideally during medical school.

This is of particular interest in Germany since reliable data are missing: Regarding the situation in Germany, Bickel et al. [10] analyzed publicly available records of 48 universities and university-associated hospitals in Germany to extract and quantify gender ratios, but did not investigate the attitudes of female medical students towards surgery. Omer et al. [11] conducted a cross-sectional survey about gender disparities and their impact on the professional experiences of 63 female neurosurgery residents in Germany, also excluding the position of female medical students. Hertling et al. [12] investigated gender ratios specifically in obstetrics and gynecology, but also do not provide insights about the perspective of female medical students and their needs. Gender bias in leadership positions in the field of plastic surgery was studied by Sadoun et al. [13] in a large study with more than 800 participants, but focussed also only on practicing physicians. In summary, the literature is focused on trainee experience instead of the perceptions of female medical students, a gap which our study closes.

The most apparent obstacles for female medical students in Germany to consider a career as a surgeon we found in our study can be summarized as follows:

- Gender discrimination presents an important obstacle. While 77.9% mark gender discrimination as a highly daunting aspect of surgery, this number drops to 47.4% of our participants who liked gender discrimination least when considering their already made experiences in internships. Even more, 62.98% name concepts against discrimination as an action which could be taken to improve interest in a career as a female surgeon. This is also reflected in 81.22% naming programs for fostering appreciative communication in the operating room as one such action. As a consequence, a future curriculum to foster interest in surgical careers must be based on anti-discrimination concepts on multiple levels, including all relevant stakeholders: Universities, academic hospitals as well as obligatory internships during medical school.
- The load of work and work times is perceived by 89.5% as highly daunting, and even 95.03% rate more flexible work times and a better work-life-balance as an important action to be taken to improve interest in a career as a female surgeon, compare also Fig. 6a, which shows that flexible work times are perceived as important or very important by the majority of female medical students. This is probably the most relevant point after anti-discrimination concepts, as there seems to be consensus between medical students that this presents a core deterrent to consider a career as a surgeon, in particular, as the majority of students rated their experiences in surgery during internships as neutral of positive, compare Fig. 4c.
- The lack of female role models and mentoring programmes is further visible by inspecting the results of our study: About three out of four students (75.98%) would participate in such a programme. 50.28% rate it as an action which could improve interest in a career as a female surgeon. 52.49% of the participants rate more available female mentors and role models as another such action. This contributes to the work environment, which was liked least by 53.76% of the participants when considering their already made experiences in internships.

### Limitations

Our study clearly has several limitations, which we outline here briefly. First, our study included only two universities in western Germany, so perceptions of female medical students in entire Germany or even internationally might differ from the ones reported here. Also, our study is not longitudinal, and it might be interesting to investigate how perceptions about surgery change over time in female medical students. This would allow to spot turning points where interest plateaus or even starts to decrease, possibly due to negative experiences during a surgical rotation or internship. However, such a study would require much more effort and was not the scope of this research, but constitutes a valuable extension in the future. Third, our three core recommendations are based on a questionnaire which was constructed based on the available literature and further items dealing with key challenges for female medical students to consider a career as a surgeon. Additional questionnaire items could provide an even richer image of the situation, even though we expect that (1) gender discrimination, (2) the load of work and work times and (3) the lack of female role models would still be among the top challenges for female medical students when analyzing the results.

## Conclusions

Our results indicate that a curriculum must be involved in surgical careers and must incorporate three core ingredients: (1) effective concepts against gender discrimination, (2) measures to work towards more flexible work times and better work-life balance of female surgeons, and (3) an increase in female role models and mentoring programs, tailored specifically for female medical students. Whether relevant stakeholders in Germany will succeed in providing a curriculum to provide these three core ingredients will, based on the results of this multicentric study, critically influence whether female medical students’ attitudes towards a possible career as a surgeon will improve or stay negative.

## Supplementary Information

Additional file 1. The original questionnaire in German is available in Supplemental file 1. Details about the construction of the questionnaire are available in Supplemental file 2.

## Data Availability

All data supporting the findings of this study are available within the paper and its Supplementary Information.
