# Supplementary material for: Contemporary challenges for a curriculum to foster interest in surgical careers: a multicentric study on the evolving needs of female medical students to consider a career as a surgeon in Germany
Source: BMC Med Educ. 2026 Jul 3;26:1070. doi: 10.1186/s12909-026-09247-y (PMC13330337; doi:10.1186/s12909-026-09247-y)
Supplement: Supplementary file 1 — Additional file 1. The original questionnaire in German is available in Supplemental file 1. Details about the construction of the questionnaire are available in Supplemental file 2. [file 12909_2026_9247_MOESM1_ESM.zip › Supplement_3.pdf]

Marketing Perspective on the Questionnaire: Why These Questions Matter and Why Women Are the Focus

1. **Target Group Focus: Why Address Women Specifically?**

- Surgery has traditionally been a male-dominated field. Research shows that women are less likely to pursue surgery due to factors such as lack of female role models, experiences of discrimination, and challenges with work-life balance.

- To develop effective strategies, it is crucial to understand the specific needs and barriers of this underrepresented group. This is why the questionnaire is aimed specifically at female medical students, who are at a critical decision-making point in their careers.

Marketing View:

As in any effective marketing approach, understanding the target audience's motivations and pain points is key to designing tailored solutions.

This initiative is a form of diversity marketing, aimed at increasing representation and enhancing the hospital's reputation as a progressive and inclusive employer.

Questionnaire Structure: Why These Specific Questions?

Age and Semester Questions:
Useful for segmenting responses based on career stage – early interest vs. practical exposure.

Gender Identification:
Enables comparisons and deeper analysis. While the focus is on women, having data on all genders helps contextualize responses.

Appealing vs. Discouraging Aspects of Surgery:
This mirrors a classic push-and-pull model:
Pull factors (what attracts): saving lives, technical challenge, prestige
Push factors (what deters): workload, discrimination, lack of female mentors.

Helps the hospital highlight the positives and directly address the negatives in their communications and HR strategies.

Mentorship and Support Measures:
These questions are about HR product development. Responses can guide the creation of targeted programs (female mentorship, flexible schedules).
Mentorship in particular is crucial – role models have been proven to influence women's career choices positively in STEM and medicine.

Practical Experience Questions:
These reflect a customer journey analysis: Who has already "tested" the product (surgery), and how did they experience it? What worked well, what didn’t?

Flexibility and Work-Life Balance:
These are key decision-making factors for many women. Insights here can guide the development of employer branding strategies such as family-friendly policies or part-time training models.

Additional Support Resources:
This open-ended section helps uncover untapped needs and ideas for further support initiatives (networking events, training programs, financial aid).
