# Supplementary material for: Contemporary challenges for a curriculum to foster interest in surgical careers: a multicentric study on the evolving needs of female medical students to consider a career as a surgeon in Germany
Source: BMC Med Educ. 2026 Jul 3;26:1070. doi: 10.1186/s12909-026-09247-y (PMC13330337; doi:10.1186/s12909-026-09247-y)
Supplement: Supplementary file 1 — Additional file 1. The original questionnaire in German is available in Supplemental file 1. Details about the construction of the questionnaire are available in Supplemental file 2. [file 12909_2026_9247_MOESM1_ESM.zip › Supplement_1.pdf]

Liebe Medizinstudentin,

wir möchten besser verstehen, wie wir die Chirurgie als Fachgebiet für Frauen attraktiver gestalten können. Deine Antworten helfen uns dabei, Maßnahmen zu entwickeln, die den Bedürfnissen und Wünschen zukünftiger Chirurginnen entsprechen. Wir danken dir für deine Teilnahme!

1. Zu welcher der nachfolgenden Alterskategorien gehörst du?

( ) 17 oder jünger

( ) 18-20

( ) 21-29

( ) 30-39

( ) 40-49

( ) 50-59

( ) 60 oder älter

2. Bitte gib dein Semester an Semester:

( ) < 3

( ) 3-6

( ) 7-9

( ) 10-12

( ) > 12

3. Bitte gib dein Geschlecht an:

( ) weiblich

( ) männlich

( ) divers

4. Welche Aspekte der Chirurgie findest du besonders ansprechend?

( ) Die Möglichkeit, Leben zu retten

( ) Die technische Herausforderung (z.B. Einsatz von Robotern)

( ) Die Vielfältigkeit der Fälle

( ) Das Prestige des Fachgebiets

( ) Das handwerkliche Arbeiten

5. Welche Aspekte der Chirurgie empfindest du als abschreckend?

( ) Arbeitsbelastung und lange Arbeitszeiten

( ) Mangel an weiblichen Vorbildern

( ) Geschlechterdiskriminierung

( ) Physische Anforderungen

6. Welche Maßnahmen könnten deiner Meinung nach dazu beitragen, mehr Frauen für die Chirurgie zu begeistern?

( ) Mehr weibliche Mentorinnen und Vorbilder

( ) Flexiblere Arbeitszeiten und bessere Work-Life-Balance

( ) Programme zur Förderung von Frauen in der Chirurgie

( ) Aufklärung über chirurgische Karrieremöglichkeiten während des Studiums

( ) Antidiskriminierungskonzepte

( ) Programme zur Förderung einer respektvollen Kommunikation im OP

( ) Unterstützungsprogramme während PJ

7. Hast du bereits praktische Erfahrungen in der Chirurgie gesammelt (z.B. Praktika, Famulaturen)?

( ) Ja

( ) Nein

8. Wenn ja, wie war deine Erfahrung?

( ) Sehr positiv

( ) Positiv

( ) Neutral

( ) Negativ

( ) Sehr negativ

9. Was hat dir bei deinen bisherigen chirurgischen Erfahrungen am meisten gefallen?

( ) Zusammenarbeit im Team

( ) Die moderne technische Ausstattung

( ) Das operative Handwerk

( ) Patienteninteraktionen

( ) Die Lernmöglichkeiten

( ) Ich habe noch keine Erfahrung gesammelt

10. Was hat dir bei deinen bisherigen chirurgischen Erfahrungen am wenigsten gefallen?

( ) Arbeitsumfeld

( ) Geschlechterdiskriminierung

( ) Arbeitsbelastung

( ) Mangel an Unterstützung

( ) Ich habe noch keine Erfahrung gesammelt

11. Wie wichtig sind dir flexible Arbeitszeiten in deiner zukünftigen chirurgischen Karriere?

( ) Sehr wichtig

( ) Wichtig

( ) Neutral

( ) Weniger wichtig

( ) Unwichtig

12. Würdest du an einem Mentoring-Programm teilnehmen, das speziell auf angehende Chirurginnen zugeschnitten ist?

( ) Ja

( ) Nein

( ) Vielleicht

13. Welche zusätzlichen Angebote und Ressourcen könnten dir helfen, eine Karriere in der Chirurgie anzustreben?

( ) Netzwerkveranstaltungen und Konferenzen

( ) Finanzielle Unterstützung für Weiterbildungen

( ) Zugang zu speziellen Kursen und Trainings

( ) Unterstützung bei der Vereinbarkeit von Beruf und Familie

( ) Andere

Vielen Dank für deine Zeit und deine wertvollen Antworten!
