# Supplementary material for: Contemporary challenges for a curriculum to foster interest in surgical careers: a multicentric study on the evolving needs of female medical students to consider a career as a surgeon in Germany
Source: BMC Med Educ. 2026 Jul 3;26:1070. doi: 10.1186/s12909-026-09247-y (PMC13330337; doi:10.1186/s12909-026-09247-y)
Supplement: Supplementary file 1 — Additional file 1. The original questionnaire in German is available in Supplemental file 1. Details about the construction of the questionnaire are available in Supplemental file 2. [file 12909_2026_9247_MOESM1_ESM.zip › Supplement_2.pdf]

Dear medical student,

We want to develop a better understanding how to make a career as a surgeon more appealing for women. Your answers can help us to build a curriculum and take actions which are in line with the requirements and needs of future female surgeons. We already thank you for your time and participation in this study!

1. To which of the following age categories do you belong?

	17 or younger
	18-20
	21-29
	30-39
	40-49
	50-59
	60 or older

2. Please mark in which semester you are enrolled right now:

	< 3
	3-6
	7-9
	10-12
	> 12

3. Please provide your gender:

	female
	male
	diverse

4. Which aspects of surgery are highly appealing to you?

	The opportunity to save a life
	The technical challenge (e.g. roboter-assisted surgery)
	The diversity of the cases
	The prestige of the field
	Working with your own hands

5. Which aspects of surgery are highly daunting to you?

	Load of work and work times

	Lack of female role models
	Gender discrimination
	Physical requirements

6. Which aspects of surgery are highly appealing to youWhich actions could be taken to improve interest in a career as a female surgeon?

	More female mentors and role models
	More flexible work times and a better work-life-balance
	Programmes for supporting females in surgery
	Information about careers as a surgeon during medical school
	Concepts against discrimination
	Programs for fostering appreciative communication in the operating room
	Support programmes during the practical year in medical school

7. Which aspects of surgery are highly appealing to youDo you already have practical experiences from internships in surgery?

	Yes
	No

8. Which aspects of surgery are highly appealing to youHow was your experience during internships in surgery?

	Very positive
	Positive
	Neutral
	Negative
	Very negative

9. What did you like most when considering your already made experiences in internships?

	Teamwork
	Modern technical equipment
	Surgery as a craft
	Interacting with patients
	The learning opportunities
	I have not yet made any experiences

10. What did you like least when considering your already made experiences in internships?

	Work environment
	Gender discrimination
	Work load
	Lack of support
	I have not yet made any experiences

11. How important are flexible work times for your future career as a surgeon?

	Very important
	Important
	Neutral
	Less important
	Not important at all

12. Would you participate in a mentoring programme which is specially designed for female students who consider a career as a surgeon?

	Yes
	No

13. Which offers and ressources would help you to consider a career as a surgeon?

	Networking events and conferences
	Financial support for continuing education
	Access to specific courses and trainings
	Support at compatibility of work and family duties
	Others

Thanks for your time and your helpful answers!
